# Knowledge and Practice of Personal Protective Measures Against COVID-19 in Africa: Systematic Review

**DOI:** 10.2196/44051

**Published:** 2023-05-16

**Authors:** Joseph Kawuki, Paul Shing-fong Chan, Yuan Fang, Siyu Chen, Phoenix K H Mo, Zixin Wang

**Affiliations:** 1 Centre for Health Behaviours Research Jockey Club School of Public Health and Primary Care The Chinese University of Hong Kong Shatin, NT China (Hong Kong); 2 Department of Health and Physical Education the Education University of Hong Kong New Territories China (Hong Kong)

**Keywords:** personal protective measures, mask use, social distancing, hand hygiene, COVID-19, Africa, nonpharmaceutical interventions

## Abstract

**Background:**

With COVID-19 being a newly evolving disease, its response measures largely depend on the practice of and compliance with personal protective measures (PPMs).

**Objective:**

This systematic review aimed to examine the knowledge and practice of COVID-19 PPMs in African countries as documented in the published literature.

**Methods:**

A systematic search was conducted on the Scopus, PubMed, and Web of Science databases using appropriate keywords and predefined eligibility criteria for the selection of relevant studies. Only population-based original research studies (including qualitative, quantitative, and mixed methods studies) conducted in Africa and published in the English language were included. The screening process and data extraction were performed according to a preregistered protocol in PROSPERO (CRD42022355101) and followed the PRISMA (Preferred Reporting Items for Systematic Reviews and Meta-Analyses) guidelines. The quality of the included studies was assessed using the Mixed Methods Appraisal Tool. Thematic analysis was used to systematically summarize the studies into 4 predefined domains: knowledge and perception of PPMs, mask use, social and physical distancing, and handwashing and hand hygiene, including their respective levels and associated factors.

**Results:**

A total of 58 studies across 12 African countries were included, published between 2019 and 2022. African communities, including various population groups, had varying levels of knowledge and practice of COVID-19 PPMs, with the lack of personal protective equipment (mainly face masks) and side effects (among health care workers) being the major reasons for poor compliance. Lower rates of handwashing and hand hygiene were particularly noted in several African countries, especially among low-income urban and slum dwellers, with the main barrier being the lack of safe and clean water. Various cognitive (knowledge and perception), sociodemographic, and economic factors were associated with the practice of COVID-19 PPMs. Moreover, there were evident research inequalities at the regional level, with East Africa contributing 36% (21/58) of the studies, West Africa contributing 21% (12/58), North Africa contributing 17% (10/58), Southern Africa contributing 7% (4/58), and no single-country study from Central Africa. Nonetheless, the overall quality of the included studies was generally good as they satisfied most of the quality assessment criteria.

**Conclusions:**

There is a need to enhance local capacity to produce and supply personal protective equipment. Consideration of various cognitive, demographic, and socioeconomic differences, with extra focus on the most vulnerable, is crucial for inclusive and more effective strategies against the pandemic. Moreover, more focus and involvement in community behavioral research are needed to fully understand and address the dynamics of the current pandemic in Africa.

**Trial Registration:**

PROSPERO International Prospective Register of Systematic Reviews CRD42022355101; https://www.crd.york.ac.uk/prospero/display_record.php?ID=CRD42022355101

## Introduction

### Background

After its emergence in December 2019, COVID-19 was declared a pandemic by the World Health Organization on March 11, 2020, and it has spread to almost all countries and regions, including Africa [1,2]. Spreading to the continent through travelers returning from hot spots in Asia, Europe, and the United States, COVID-19 was first recorded in Africa in Egypt on February 14, 2020, and within a few months, the virus had spread throughout the continent [1,3]. As of March 20, 2023, a total of 12,804,191 cumulative cases and 258,623 deaths have been reported in Africa compared with 682,546,389 cases and 6,819,835 deaths across the globe, showing a disproportionately low case fatality rate of COVID-19 in Africa [4].

As in the rest of the world, various response measures were implemented in different African countries to curb the spread of the virus, including statewide lockdowns, restrictions on movement, bans on social gatherings, and school closures [5,6]. Although the continent appears to have a lower absolute number of cases and deaths compared with other regions [7], which might also be related to the lower number of tests administered, the pandemic has had a deep impact on the socioeconomic systems of African countries [8,9]. The pandemic has also strained the weak and fragmented health systems, as shown by the lack of personal protective equipment (PPE), testing kits, and other treatment necessities, especially for patients with COVID-19 who are critically ill [6,8].

With COVID-19 being a newly evolving disease, its less-defined outcomes and unprecedented prevention, treatment, and control modes largely require indisputable collaboration among various stakeholders in the community [9]. Nonpharmaceutical interventions play an important role in the control and prevention of pandemics, including the COVID-19 pandemic, especially in its early phase and wave. Despite the availability of approved vaccines against COVID-19, response measures toward this pandemic still largely depend on the practice of and compliance with personal protective measures (PPMs), including face mask use, social and physical distancing, and hand hygiene [10]. Moreover, knowledge and perceptions of PPMs have been reported as among the key determinants of practice and compliance with PPMs against COVID-19 as they influence people’s behavior [10,11]. These were also considered in this study in the African context.

The pandemic has had a broad range of impacts and challenges across regions, and different communities have responded differently. However, given the diversity of social systems across regions and countries, preparedness and the search for a country- or region-specific practical solution to the pandemic require a better understanding of the challenges of practicing PPMs and hard-learned experiences through comprehensive research [9]. There has been a high research output documenting COVID-19 characteristics, clinical outcomes, response, and impact throughout the world but with much less research coming from African countries [12,13]. The unavailability of research information might be seen as a barrier to successful prevention and further as a sign of inequity between high- and low-income countries and regions [14]. This scant literature poses knowledge gaps on how African countries are responding to the pandemic in terms of PPMs. Nonetheless, a recent review by Nwagbara et al [15] reported that most communities in sub-Saharan Africa had a positive attitude toward and good practices regarding COVID-19. Notably, this review considered studies only from sub-Saharan Africa and those conducted in the first stages of the pandemic, so it lacked insights into the overall practice of PPMs in Africa.

### Objectives

Thus, this systematic review aimed to examine COVID-19 PPM research from African countries as documented in the published literature. On the basis of specific keywords, the review looked at the levels and associated factors of (1) knowledge and perception of PPMs and (2) practice of COVID-19 PPMs in various populations (including face mask use, physical and social distancing, and handwashing and hand hygiene).

## Methods

### Study Design

This systematic review was conducted according to a preregistered protocol in PROSPERO (CRD42022355101) and the PRISMA (Preferred Reporting Items for Systematic Reviews and Meta-Analyses) guidelines (Multimedia Appendix 1) [16]. This systematic review considered literature concerning PPMs from African countries. Literature was sourced from the following databases: Scopus, PubMed, and Web of Science. These databases were considered as they sufficiently cover most of the key journals, including most African journals. In addition, 2 of these databases (Scopus and Web of Science) could refine the search based on countries and regions, unlike other databases, which enabled us to specifically assess publications from African countries only.

### Search Strategy

We conducted a comprehensive search using a set of appropriate keywords and Medical Subject Heading terms to identify studies reporting on PPMs. For consistency and precision, similar keywords and Medical Subject Heading terms were used and searched for in the article titles across the databases. A comprehensive search of the published literature was performed in each of the 3 selected databases using combinations of key terms and Boolean operators (Textbox 1). These included “mask,” “nose covering,” “personal protective equipment,” “handwashing,” “hand washing,” “hand sanitizer,” “hand sanitiser,” “sanitation,” “hygiene,” “social distance,” “social distancing,” “physical distance,” “physical distancing,” “social acceptance,” “COVID-19,” “2019-nCoV,” “coronavirus disease,” “SARS-CoV-2,” and “corona virus disease 2019.”

**Key terms or Boolean operators used for the search.**

- “Mask” OR “nose covering” OR “personal protective equipment” OR “handwashing” OR “hand washing” OR “hand sanitizer” OR “hand sanitiser” OR “sanitation” OR “hygiene” OR “social distance” OR “social distancing” OR “physical distance” OR “physical distancing” OR “social acceptance” AND “COVID-19” OR “2019-nCoV” OR “coronavirus disease” OR “SARS-CoV-2” OR “corona virus disease 2019”
- “Mask” OR “nose covering” OR “personal protective*” OR “hand wash*” OR “hand-wash*” OR “hand sanitize*” OR “hand sanitise*” OR “sanitation*” OR “hygiene*” OR “social distance*” OR “physical distance*” OR “social accept*” OR “social acceptance” AND “COVID-19” OR “COVID*”
- “Mask” OR “nose covering” OR “personal protective*” OR “hand wash*” OR “hand-wash*” OR “hand sanitize*” OR “hand sanitise*” OR “sanitation*” OR “hygiene*” OR “social distance*” OR “physical distance*” OR “social accept*” OR “social acceptance” AND “coronavirus*” OR “corona*”
- “Mask” OR “nose covering” OR “personal protective*” OR “hand wash*” OR “hand sanitize*” OR “hand sanitise*” OR “sanitation*” OR “hygiene*” OR “social distance*” OR “physical distance*” OR “social accept*” OR “social acceptance” AND “SARS-CoV-2*” OR “2019-nCoV*”

### Inclusion and Exclusion Criteria

The inclusion and exclusion criteria are listed in Table 1. Only population-based original research studies (including qualitative, quantitative, and mixed methods studies) conducted in Africa, published in English, and reporting on PPMs against COVID-19 were considered in the full review. In addition, multicountry studies were considered if they included an African country as part of their study population. Only English-language articles published between November 1, 2019, and March 4, 2022, were considered.

**Table 1:** Summary of the inclusion and exclusion criteria.

Parameter	Inclusion criteria	Exclusion criteria
Article or study type	Population-based original research studies Qualitative, quantitative, and mixed methods studies Multicountry studies	Reviews, commentaries, and editorials Dissertations, government reports, newspaper articles, textbooks, book chapters, and protocols Gray literature and preprints Laboratory studies, model and framework studies, and validation studies
Language	English language	All other non-English languages
Publication period	November 1, 2019, to March 4, 2022	All periods outside November 2019 to March 2022
Study setting	African countries	All non-African country settings

### Data Extraction

After screening, data from the relevant studies were independently extracted by 2 reviewers (JK and PSC) onto a structured data extraction template, and a consensus was reached through discussion in case of disagreements on the extracted data. The following variables were extracted: first author, year of publication, study location, study design, key measurements, study population, sample size. and main findings.

### Quality Assessment and Analysis

We assessed the information from the included articles using the Mixed Methods Appraisal Tool (version 2018) with detailed descriptions of the rating [17]. In total, 2 reviewers also independently assessed the quality of the included studies, and in case of discrepancies, a consensus was reached through discussion.

This study used thematic analysis, and the literature in this review was used to understand the practice of PPMs against COVID-19 in African countries. The studies were classified according to four main themes: (1) knowledge and perception of PPMs, (2) mask use, (3) social and physical distancing, and (4) handwashing and hand hygiene, including their respective levels or prevalence and associated factors. In addition, various population groups and challenges faced in practicing COVID-19 PPMs were examined under each of the main themes based on the available information in the analyzed studies. The 4 themes were drafted by a panel of public health experts after a series of discussions to reach a consensus.

The analysis process involved a six-step data synthesis process: (1) in total, 2 reviewers (JK and PSC) extracted relevant information on knowledge and practice of PPMs from each article independently; (2) after extraction, they discussed to reach a consensus on the key information identified in each article; (3) the extracted information was coded under the 4 predefined domains by the 2 reviewers independently; (4) after completing the coding independently, they discussed the results, where any discrepancies were resolved through discussion; (5) the revised coding results were read and checked by the 2 reviewers independently to ensure that all the extracted information was mapped to the 4 domains correctly; and (6) all the information in the codebook was adapted into a tabular format.

## Results

### Selection of Studies Conducted in Africa

**Figure 1:**
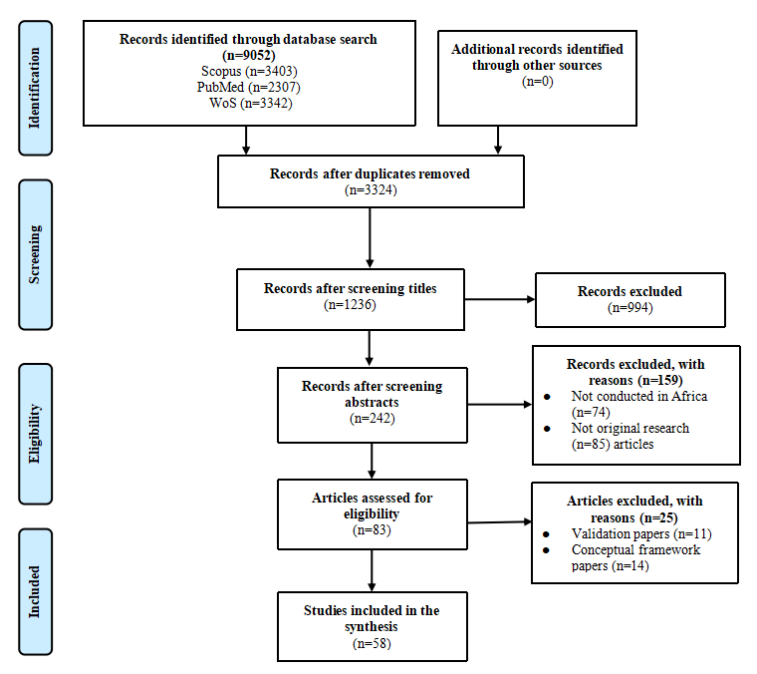
The PRISMA (Preferred Reporting Items for Systematic Reviews and Meta-Analyses) flowchart of the selected studies. WoS: Web of Science.

The number of studies identified, reviewed, and selected, including the reasons for exclusion, is summarized in Figure 1. A total of 58 studies were selected through this process and further analyzed [18-75]. The information and main findings extracted from all included studies is detailed in Table 2.

**Table 2:** Characteristics of the included studies.

Study	Study setting or country	Study type and key measures	Study population	Sample size, N	Adherence or noncompliance rates	Relevant findings
Sikakulya et al [18], 2021	Uganda	Cross-sectional; knowledge, attitudes, and practices regarding proper use of face masks	Community	1114	51.5% had poor mask use	Most participants (60.1%) had satisfactory knowledge of the use of face masks, and this was greater among participants with tertiary educational levels. Regarding attitude, 69.4% were confident enough to correctly put on a face mask, 83.4% believed that a face mask can protect against COVID-19, and 75.9% had never shared their face mask. Most (95.2%) agreed that wearing face masks in public places was important to protect themselves against COVID-19, and 60.3% reported washing their hands before wearing and after removing the face mask.
Hailu et al [20], 2021	Ethiopia	Cross-sectional and mixed methods; compliance with social distancing	Community	401	Overall, 55.4% had poor compliance with social distancing measures	Most (63.84%) reported that they went to crowded places without putting on a face mask, but 60.6% and 76.3% had good knowledge of COVID-19 transmission and prevention, respectively. Only age was associated with social distancing measures, with older persons more likely than younger persons to comply with social distancing guidelines.
Bakry and Waly [21], 2020	Egypt	Cross-sectional; perception and practice of social distancing	Community	1036	82% were not strictly practicing social distancing	Most (70%) perceived that social distancing measures reduced the transmission of COVID-19. There was a significant association between the practice of social distancing and some sociodemographic factors such as sex, age, education, working status, place of residence, and community of residence.
Tadesse et al [22], 2020	Ethiopia	Cross-sectional; predictors of preventive practices	Community employees	628	68.8% had poor COVID-19 prevention practice	Most (>50%) had high perceived susceptibility, severity, benefit, barriers, cues to action, and self-efficacy regarding COVID-19 prevention practice. Employees with a low level of perceived barriers were less likely to have a poor practice of COVID-19 prevention compared with employees with a high level of perceived barriers. Moreover, employees with low cues to action and employees with a low level of self-efficacy practiced COVID-19 prevention measures to a lesser extent compared with those with high cues to action and high levels of self-efficacy.
Iyamu et al [23], 2022	MCP^a^—6 countries: Botswana, Kenya, Malawi, Nigeria, Zambia, and Zimbabwe	Cross-sectional; face mask use perception and social media	Community	1988	—^b^	A total of 58.8% used social media as their main source of information, whereas 85% agreed that face masks were effective against COVID-19. Respondents who used social media were more likely to agree that face masks were effective compared with those who did not.
Bukuluki and Kisaakye [24], 2021	Uganda	Cross-sectional; face mask wearing in public places	Community	1054	52% and 78% wore face masks sometimes inside in public spaces and always outside in public spaces, respectively	Approximately 90% of respondents agreed that wearing a mask inside or outside in public spaces can prevent COVID-19 infection. Age and frequency of face mask wearing inside or outside in public spaces were significantly related to belief in face mask efficacy.
Nnama-Okechukwu et al [25], 2020	Nigeria	Qualitative study; knowledge of and compliance with preventive measures	Community	36	—	Findings revealed that most of the respondents believed that the COVID-19 pandemic was more of a hoax than a reality. Other findings showed that this poor knowledge negatively affected their compliance with preventive measures to curb the spread of coronavirus.
Kajiita and Kang’ethe [26], 2021	8 African countries	Cross-sectional and qualitative; social distancing perceptions	Community	20	—	Results revealed varied conceptualizations and interpretations of the disease and social distancing. Notably, COVID-19 regulations such as social distancing and face mask wearing were perceived as an imported policy, a misconception responsible for nonadherence to COVID-19 protocols. Furthermore, the study underscored that the disease and policies related to it disrupted ways of social life, infringed on people’s social-cultural rights, and had adverse health consequences.
Fodjo et al [27], 2020	10 countries; DRC^c^, Uganda, Mozambique, and Somalia	Multicountry web-based survey; compliance with mask use	General public	206,729	Face mask use—DRC: 43.2%; Uganda: 32.7%; Mozambique: 93.9%; Somalia: 51.2%	Adherence rates were higher in countries where masking was mandatory or highly encouraged by the government during the early phases of the COVID-19 outbreak. Reusable cloth masks (more cost-beneficial and environmentally friendly than surgical masks) were the most frequent, accounting for 51.1% of all mask types. There were differential rates of mask uptake and use between sexes and age groups observed in different countries. Even in countries where no preexisting culture of mask use existed, high uptake of mass masking was feasible.
Sewpaul et al [28], 2021	South Africa	Cross-sectional; compliance with and determinants of social distancing	Community	17,563	20.3% reported having not left home	A total of 50.6% were in close physical distance with 1-10 people, 21.1% were in close physical distance with 11-50 people, and 8% were in close physical distance with >50 people. Larger household sizes and incorrect knowledge about the importance of social distancing were associated with being in contact with >50 people. Male sex, younger age, and being in the White and non-White population groups were significantly associated with being in contact with 1-10 people but not with larger numbers of people. Employment, at least a secondary school education, the lack of self-efficacy in being able to protect oneself from infection, and moderate or high risk perception of becoming infected were all associated with increased odds of close contact with 1-10, 11-50, and >50 people relative to remaining at home.
Wondimu et al [29], 2020	Ethiopia	Cross-sectional; predictors of preventive practices	Community	803	Generally, 59.4% had good prevention practices for COVID-19	Approximately 64.7% had a history of going to crowded places, whereas only 30.3% of the participants had a history of wearing a mask when leaving home. A total of 64.4% had a history of maintaining their distance at 2 meters, and 64.8% washed their hands with soap and water or used alcohol-based hand sanitizers. Urban residence, family size, good knowledge, positive attitude, intention to seek care, and perceived mortality were positively associated with good prevention practices.
Fikrie et al [30], 2021	Ethiopia	Cross-sectional; social distancing and associated factors	Community	410	38.3% had good social distancing practices	Younger age (26-35 years) and being employed were positively associated with good social distancing practice, whereas poor knowledge, negative attitude, and low perceived susceptibility had a negative association.
Mejjad et al [31], 2021	Morocco	Cross-sectional; mask use and disposal behavior	Community	185	70% used face masks at least once a day	A total of 70% of the respondents threw their discarded masks and gloves in the house trash or trash bins after their first use, whereas nearly 30% of respondents admitted that they did not wear masks as they did not leave their homes during the lockdown.
Burger et al [32], 2022	South Africa	Longitudinal survey; predictors of mask wearing	Community	7074	74% wore face masks when in public	Self-efficacy, the prevalence of others’ mask wearing in the same district, and affluence were positively associated with reported mask wearing. Those who reported staying at home were significantly less likely to report wearing a mask. Despite having a higher mortality risk, older adults had significantly lower odds of mask wearing. The prevalence of mask wearing increased significantly from May 2020 to August 2020 (from 50% to 74%) as COVID-19 cases increased and lockdown restrictions were eased, but staying at home, physical distancing, and social distancing decreased.
Amuakwa-Mensah et al [33], 2021	MCP—12 sub-Saharan African countries	Cross-sectional; handwashing and COVID-19 concerns	Community	4788	54.6% washed their hands for 20 seconds >5 times a day, and 4.2% did not wash their hands at all	The level of concern about the spread of the virus increased the likelihood of washing hands with soap under running water for a minimum of 20 seconds at least 5 times a day. Heterogeneous effects across gender and age groups, locality, and various water sources were noted.
Szczuka et al [19], 2021	MCP—Gambia	Observational study; handwashing adherence	Community	6064	—	Higher handwashing adherence was associated with more frequent exposure to handwashing guidelines, being a health care professional, being older, being female, and being married. Stricter containment and health policies were associated with lower handwashing adherence.
Iwuoha and Aniche [34], 2020	Nigeria	Cross-sectional and qualitative; impact of physical distancing policies	Slum residents	49	—	The study demonstrated that the peculiar and adverse conditions of low-income urban dwellers were not considered in the formulation of the COVID-19 lockdown and physical distancing policies in Nigeria. Thus, such policies worsened the living conditions of extremely low-income urban or suburban slum dwellers in Nigeria. There is a need to engender an indigenous (Afro-centered) approach to the containment of the pandemic.
McCreesh et al [35], 2021	South Africa	Longitudinal survey; impact of social distancing regulations	Community	1704	—	Extrahousehold social contact fell substantially following the imposition of COVID-19 distancing regulations in that there were substantial declines in close physical and conversational contacts and also in beyond-household sharing of indoor space. However, there was ongoing contact within intergenerational households, highlighting a potential limitation of social distancing measures in protecting older adults.
De Backer et al [36], 2020	MCP—38 countries; Uganda, South Africa, and Egypt	Cross-sectional; impact of social distancing on healthy meals	Community	37,207	—	Increases in planning, selecting, and preparing healthy foods were found for women and men and were positively related to perceived time availability and stay-at-home policies. Psychological distress was a barrier for women and an enabler for men, whereas financial stress was a barrier and enabler depending on various sociodemographic variables.
Kim et al [37], 2022	Kenya	Cross-sectional; WASH^d^ accessibility	Slum dwellers	647	—	A total of 77.4% and 60.6% of people living in Kibera and Mathare, respectively, had limited WASH facility accessibility or opportunity. Overall accessibility and opportunity were better in Mathare than in Kibera.
Ag Ahmed et al [38], 2021	Mali	Qualitative study; adoption of physical distancing measures	Internally displaced people	68	—	The main challenges concerning the implementation and adoption of physical distancing measures included the proximity in which internally displaced people live, their beliefs and values, the lack of toilets and safe water on sites, internally displaced people’s habits and economic situation, humanitarian actors’ lack of financial resources and authority, and social pressure from religious leaders. Implemented mitigation measures included the building of new shelters or their compartmentalization, the creation of income-generating activities and food banks, psychosocial support, promotion of awareness of internally displaced people, and night police patrols and surveillance to discourage internally displaced people from going out.
Mhlanga-Gunda et al [39], 2022	Zimbabwe	Qualitative study; social distancing and prevention measures	Prisoners and staff	80	—	Outdated infrastructure, severe congestion, interrupted water supply, and inadequate hygiene and sanitation were conducive to ill health and the spread of disease. Health professionals had been well trained regarding COVID-19 control measures, and COVID-19 awareness among prisoners was generally adequate. There was no routine COVID-19 testing in place beyond thermal scanning. Access to health care was good, but standards were hindered by inadequate medicine and PPE^e^ supply, and isolation measures were compromised by accommodation capacity issues. The flow of prison entries constituted a transmission risk, and social distancing was impossible during meals and at night.
Assefa et al [40], 2021	Ethiopia	Cross-sectional; knowledge, attitude, practice, and challenges regarding hand hygiene	HCWs^f^	96	76% had good hand hygiene practices with alcohol-based hand sanitizers	All the HCWs practiced different COVID-19 prevention methods, and most were knowledgeable (93.8%) and had a favorable attitude (74%). However, 84.5% of the respondents faced challenges during alcohol-based hand sanitizer use owing to it being unavailable (68.8%) or expensive (52.1%); forgetting (11.5%); and experiencing health-associated risks such as skin irritation (28.1%), skin dryness (62.5%), and ocular irritation (11.5%).
Seid Yimer and Gebrehana Belay [41], 2021	Ethiopia	Hospital-based cross-sectional study; knowledge and practice of proper face mask use	HCWs	422	59.5% practiced proper mask use	The overall good knowledge score of health care providers regarding proper face mask use was 65.8%. Of them, 67.3% knew that face masks were worn with the white side facing in, 62.6% knew that face masks had 3 layers, and 78.4% knew that surgical face masks were worn for up to 8 hours.
Ahmed Sayed et al [42], 2021	Egypt	Cross-sectional; preparedness and attitude toward PPE	HCWs	254	—	Only 28.74% of the house officers had good preparedness, whereas 85.83% had a good PPE attitude. Preparedness and willingness were significantly associated with the overall worry related to the pandemic (fear of contracting COVID-19 and having family members at risk of severe COVID-19). The house officers with good preparedness and willingness to deal with COVID-19 seemed to have a good PPE attitude.
Alao et al [43], 2020	Nigeria	Cross-sectional; knowledge, attitudes, beliefs, and use of PPE	HCWs	272	—	Only 25.7% had adequate knowledge about PPE. Of the respondents who presumed that they had adequate knowledge about donning and doffing PPE, 94 (56%) were incorrect. The predictors of good knowledge were younger age (<45 years) and practice location.
Daghmouri et al [44], 2020	Tunisia	Cross-sectional and institution-based; PPE use	HCWs	723	—	There was a likely overuse of PPE in addition to a high rate of side effects caused by PPE. A total of 57.3% of participants reported a lack of PPE, which could be extremely stressful and detrimental to them. A total of 72.5% of respondents reused disposable PPE, especially facial protective shields and FFP2^g^. Only 37.8% of frontline HCWs had received official training on the correct use of PPE, especially on how to fit FFP2 masks (only 32.6%). A total of 89.1% of participants believed that they needed additional training.
Kassie et al [45], 2020	Ethiopia	Cross-sectional; preventive practices	HCWs	630	38.7% (95% CI 34.8%-42.5%) good preventive practice against COVID-19	Being a male health care provider, having work experience of 6-10 years, and having a poor attitude toward COVID-19 were found to be significantly associated with poor COVID-19 preventive practices among HCWs.
Keleb et al [46], 2021	Ethiopia	Cross-sectional; PPE use and hand hygiene and associated factors	HCWs	489	32% and 22.3% were compliant with PPE use and hand hygiene practice, respectively	Feedback for safety, training on COVID-19 prevention, and perception of infection risk were significant factors of good compliance with PPE use.
Birhanu et al [47], 2021	Ethiopia	Cross-sectional; PPE use	HCWs	418	37.6% had good practice of PPE use	Being male, being a nurse or midwifery professional, regularly sanitizing hands and medical equipment, having national COVID-19 management guidelines, taking COVID-19 training, and the feeling of eventually contracting COVID-19 at the workplace had a positive association with PPE use. However, not feeling safe at work when using standard precautions was negatively associated with PPE use.
El-Sokkary et al [48], 2021	Egypt	Cross-sectional; mask use and compliance	HCWs	404	53.2% were noncompliant with mask use	Most reported a shortage in N95 respirators (91.3%) and practiced extended PPE use (88.1%). Better compliance with proper PPE use was reported among female individuals, physicians, and medical specialties with <10 years of work experience and working >8 hours per day. Significant predictors of compliance were receiving previous training on the proper use of PPE, exposure to patients with COVID-19, and performing procedures that posed a high risk of exposure to COVID-19 to HCWs.
Afemikhe et al [49], 2020	Nigeria	Cross-sectional; transmission-based precaution practices	Nurses	367	85.6% maintained a good level of preventive practices, and 89.1% performed hand hygiene	Academic qualification was a significant predictor of good practice in favor of respondents with a degree in Nursing. Challenges identified were the lack of financial motivation, fear of infecting family members, and fear of contracting the virus (93.5%).
Elhadi et al [50], 2021	Libya	Cross-sectional; mask wearing	General population and HCWs	15,087	68.1% had mask wearing adherence	—
Tabah et al [51], 2020	90 countries; Libya, Egypt, Morocco, and Tunisia	A cross-sectional, international survey; PPE use	HCWs	2711	For routine care, 58% used FFP2 or N95 masks, waterproof long-sleeved gowns (67%), and face shields or visors (62%)	Powered air-purifying respirators were used routinely and for intubation only by 7% and 13% of respondents, respectively. Surgical masks were used for routine care and for intubations by 15% and 2% of respondents, respectively. At least one piece of standard PPE was unavailable for 1402 (52%) respondents, and 30% reported reusing single-use PPE. PPE was worn for a median of 4 (IQR 2-5) hours. Adverse effects of PPE were associated with longer shift durations and included heat (51%), thirst (47%), pressure areas (44%), headaches (28%), inability to use the bathroom (27%), and extreme exhaustion (20%).
Mahmoud et al [52], 2021	Egypt and Saudi Arabia	Comparative and cross-sectional; effect of sanitizers and PPE use	HCWs	428	—	The most affected areas from wearing PPE were the hands (49.8% and 54.5%), followed by the auricular area (44% and 40.9%), the nasal bridge (28.9% and 22.7%), the cheeks (16.9% and 13.6%), and the whole face (15.6% and 25%) among Saudi and Egyptian HCWs, respectively. Approximately 70% of Egyptian HCW participants used 70% alcohol in the form of a gel as sanitizer, which was significantly higher than Saudi HCWs (59.1%). The most adverse reaction to using sanitizers was skin dryness (55.1% and 63.6% among Saudi and Egyptian HCWs, respectively).
Shadi et al [53], 2022	MCP that included Egypt	Cross-sectional; PPE use and hand hygiene	HCWs	154	66.9% used N95, N98, or a surgical mask, and 86.4% had good hand hygiene	A total of 77.3% had all the PPE and protective measures they needed. A total of 66.2% had been recently educated on infection control. A total of 20.8% always used a standard surgical mask and thought that it was sufficiently protective, 12.3% used either mask according to their availability, and none of the participants refrained from using masks.
Deressa et al [54], 2021	Ethiopia	Cross-sectional; availability and use of PPE and satisfaction with PPE	HCWs	1134	—	Most (77%) of the HCWs reported that their hospital did not have adequate PPE. A critical shortage of N95 respirators was particularly reported; it only increased from 13% to 24% from before to during the COVID-19 pandemic. The use of N95 increased from 9% to 21% from before to during the COVID-19 pandemic. Almost 72% of the respondents were dissatisfied with the availability and use of PPE in their hospital. The independent predictors of the respondents’ satisfaction levels with PPE were HCWs who reported that PPE was adequately available in the hospital and preparedness to provide care to COVID-19 cases.
Oladele et al [55], 2021	Nigeria	Cross-sectional and mixed methods; availability and use of PPE	HCWs	258	—	Only 22.1% of HCWs had regular access to PPE, and only 20.6% had access to N95 face masks compared with other PPEs. Male HCWs and those working at secondary or tertiary facilities had access to N95 face masks. Facilitators of PPE use were the leadership quality of the hospital head and donation of PPE to the facilities, whereas barriers to PPE use included a limited supply of PPE as well as the facility’s infrastructural and operational challenges.
Ashour et al [56], 2021	MCP—Egypt and Morocco	Cross-sectional; challenges and difficulties of using PPE	Ophthalmologists	172	—	The analysis of the responses showed that most ophthalmologists used face masks without substantial problems during their examinations, whereas face shields followed by protective goggles were the most inconvenient PPE in the current ophthalmic practice. Moreover, most (77.3%) noticed an increase in their examination time when using PPE. A considerable proportion (40.7%) stopped using one or more of the PPE because of inconvenience or discomfort.
Foula et al [57], 2021	Egypt	Cross-sectional; effect of wearing PPE on performance and decision-making	Physicians	272	—	Results indicated that comfort, vision, and communication were significantly reduced because of PPE wearing in all physician groups (81.1%, 88.7%, and 75.5%, respectively). In contrast, the handling of instruments was not significantly affected in the second group only. Moreover, decision-making and the rate of complications were not significantly affected.
Hajjij et al [58], 2020	Morocco	Cross-sectional; PPE and headaches	HCWs	155	—	The overall prevalence of headaches related to PPE was 62%. It was experienced de novo by 32.9% of participants, whereas it was an aggravation of a preexisting headache in 29% of participants. Working >8 hours per shift during the pandemic was correlated to de novo headache (*P*=.008). The profession of physician and working >12 hours per shift were correlated with aggravated headaches. HCWs experienced moderate discomfort, blurred vision, and reduced concentration. They judged their professional performance to be mildly reduced by the use of PPE.
Nwosu et al [59], 2021	Nigeria	Cross-sectional; impact of different face masks on comfort	HCWs	66	—	HCWs wore masks for periods ranging from 68 to 480 minutes. The discomfort experienced with the use of the N95 mask was greater than with the surgical mask. No significant change in arterial oxygen saturation was observed with the use of either mask type, and the tight strapping of the N95 mask was perceived as a contributor to the discomfort experienced with mask use.
Marraha et al [60], 2021	Morocco	Cross-sectional; skin reactions to PPE use	HCWs	273	—	A total of 80% of HCWs had adverse reactions, including skin problems, after wearing goggles (58%), wearing surgical masks and respirators (57%), handwashing and wearing gloves (45%), wearing a face shield (23%), and wearing protective clothing (11%). Bleach immersion was highly significantly associated with hand reactions, whereas hand cream use more than twice daily was associated with fewer reactions. The skin reactions were related to goggle use, wearing masks and N95 respirators was significantly associated with longer use duration, and adverse reactions to regular use of protective clothing were related to the frequency of its use per shift.
Jazieh et al [61], 2020	6 countries; Egypt, Algeria, and Morocco	Multicountry survey; behavioral response	Patients with cancer	1012	Adherence to handwashing (77%), keeping distance from others (67%), mask use (77%), and hand hygiene with hand sanitizer (69%) and soap (81%)	Patients were worried about contracting the virus strongly (33%) or mildly (48%), and most (>80%) reported avoiding the following actions: hand shaking, hugging and kissing, social gatherings, meeting friends, and visiting markets. Some reported adopting healthier diets (35%), using dietary supplements (18%), and reciting the Quran (61%) or supplications (75%). Approximately 23% would choose not to show up for a scheduled medical appointment, and 43% had appointment cancellations at the request of the medical team (31%) or the patients themselves (12%). Moreover, 84% preferred web-based medical appointments over regular visits.
Andarge et al [62], 2020	Ethiopia	Cross-sectional and facility-based; intention and practice of PPMs^h^	Adults with chronic conditions	806	52% and 76.3% intended to practice and had ever practiced PPMs	Participants’ subjective norms and perceived behavioral control were the factors associated with their intention. Good knowledge and a positive attitude were found to be significant factors associated with the participants’ actual practice of PPMs among other independent factors.
Mostafa and Hegazy [63], 2020	Egypt	Cross-sectional, observational study	Patients of dermatology	62	—	There was an overall satisfaction and future use score of 91% among the interviewed patients who received teledermatology services; a usefulness score of 93.7%; interface and interaction quality scores of 85.9% and 87%, respectively; ease and use learnability score of 87.8%; and a reliability score of 86.7%.
Larebo and Abame [64], 2021	Ethiopia	Cross-sectional; face mask use and associated factors	University students	764	89.5% had good practice of face mask use	Overall knowledge of the students was 29.2%, and their attitude was 88.1%. Students from the College of Natural and Computational Sciences and students having good knowledge were found to be independently associated with face mask use.
Nalunkuma et al [65], 2022	Uganda	Cross-sectional; patterns of double mask use	Medical students	348	Only 20.5% reported double masking	A total of 68.7% believed that double masking was superior to single masking for COVID-19 prevention and control. Those with a past COVID-19 positive test and those who believed that double masks had a superior protective advantage were more likely to double mask. The lack of trust in the quality of masks (46.5%) was the most frequent motivation for double masking, whereas excessive sweating (68.4%), high cost of masks (66.4%), and difficulty in breathing (66.1%) were the major barriers.
Aronu et al [66], 2020	Nigeria	Cross-sectional; perception of masking in children	Mothers	387	—	Only 44.7% of the mothers perceived masking in children as an appropriate measure for the prevention of COVID-19, and the frequent reasons for the inappropriateness of face masks in children given by most (55.3%) of the mothers included perceived difficulty in breathing (38.5%) and the child’s readiness to take the masks off (29.3%). A significantly higher proportion of children whose mothers were aged ≥35 years would wear face masks (64.2%) when compared with 31.7% of those whose mothers were aged <30 years. Similarly, 51% of the children who were aged >1 year would wear a face mask compared with 20.5% of those aged 8 days to 1 year. The children whose mothers were aged <30 years were approximately 4 times less likely to wear a face mask when compared with those whose mothers were aged ≥35 years. The children whose fathers had attained tertiary education were approximately twice less likely to wear face masks when compared with those whose fathers had attained a secondary education or lower.
Haftom and Petrucka [67], 2021	Ethiopia	Cross-sectional; face mask use	Quarantined adults	331	46% did not wear a face mask when leaving home	Face mask use was significantly associated with the knowledge score, employment status, gender, age, and educational status of the study participants.
Deressa et al [68], 2021	Ethiopia	Cross-sectional; social distancing and preventive measures	Government employees	1573	96% wore face masks, 94.5% practiced frequent handwashing, and 89.5% practiced physical distancing	A total of 94.8% avoided close contact with people, including hand shaking; 95.6% consistently followed government recommendations; 88.1% avoided mass gatherings and crowded places; 71.8% restricted movement and traveling; and 35.6% stayed home. A total of 80% perceived that consistently wearing a face mask was highly effective in preventing coronavirus infection, and the perception varied by region (Oromia residents being less likely to have good perceptions). A total of 57% perceived that the policy measures in response to the pandemic were inadequate.
Dzisi and Dei [69], 2020	Ghana	Cross-sectional, roadside observer survey; adherence to social distancing and mask use	Commuters	850	98% of buses complied with the social distancing guidelines	The policy on face masks was complied with only partially in most vehicles. A total of 12.6% of the vehicles had <3 commuters without face masks, whereas 21.3% of buses had <3 people with face masks.
Agyemang et al [70], 2021	Ghana	Cross-sectional; perception and mask use	Commercial drivers	500	—	Most drivers had a high vulnerability perception to COVID-19. It further emerged that older drivers in particular consistently wore face masks and insisted on other persons in their commercial vehicles to follow suit. Sociodemographic factors and the need to ensure one’s safety and that of loved ones were critical determinants of face mask use among surveyed drivers.
Natnael et al [71], 2021	Ethiopia	Cross-sectional; knowledge, attitude, and frequent hand hygiene practices	Taxi drivers	417	66.4% had good frequent hand hygiene practices	Good knowledge and positive attitude were reported in 69.8% and 67.6% of the drivers, respectively. Educational level, place of residence, and attitude toward COVID-19 prevention were factors associated with good knowledge about COVID-19. Furthermore, age of >30 years, a secondary education or higher, income, and knowledge about COVID-19 in taxi drivers were factors associated with a positive attitude toward COVID-19 prevention. Moreover, attitude toward COVID-19 and educational level were the factors associated with good frequent hand hygiene practices.
Mboowa et al [72], 2021	Uganda	Cross-sectional; knowledge, attitudes, and practices regarding face mask use	High-risk groups	644	—	Most had heard about COVID-19 (99.7%) and believed that face masks were protective against it (87.3%), whereas 67.9% reported having received information on face mask use. Food market vendors and those with no formal education were 0.5 and 0.3 times less likely to have received information about face mask use than hospital workers and those who had completed secondary school, respectively. Those who had received information on face mask use were 2.9 and 1.8 times more likely to own face masks and perceive them as protective, respectively. Food market vendors were 3.9 times more likely to reuse their face masks than hospital workers.
Fielmua et al [73], 2021	Ghana	Cross-sectional, observational study; hand hygiene and safety behaviors	Shoppers and shopkeepers	751	91.3% of the customers did not practice handwashing, and 84.2% did not wear face masks	It was observed that adherence to COVID-19 safety protocols at shopping centers was very poor, and in 78% of the shops observed, no shop attendant wore a mask. Despite the provision of handwashing facilities and widespread advocacy to minimize COVID-19 infections, the citizenry, especially the youth, demonstrated a poor attitude toward safety measures. Nonadherence to COVID-19 protocols was higher in shops where there was no pressure to conform to the protocols.
Ameme et al [74], 2021	Ghana	Observational study; hand hygiene and face mask wearing practices	Shop patrons	800	81.6% wore face masks, 12.3% performed hand hygiene, and 11.5% adhered to both measures	A total of 72.3% of patrons wore face masks appropriately, whereas appropriate handwashing was recorded among only 10.1%. Compared with inappropriate handwashing, appropriate handwashing was negatively associated with adherence to infection and control guidelines.
Yigzaw et al [75], 2021	Ethiopia	Observational cross-sectional study; handwashing practice	Bank visitors	415	—	Most (93.5%) heard and watched proper handwashing practice. The proportion of proper handwashing performance was 21.4% before the demonstration, but after the demonstration, it increased to 82.2%. Older age, being married, and higher education were associated with proper handwashing practice. Overall, there was a significant change in handwashing practice after the demonstration.

^a^MCP: multicountry paper.

^b^Not available.

^c^DRC: Democratic Republic of the Congo.

^d^WASH: water, sanitation, and hygiene.

^e^PPE: personal protective equipment.

^f^HCW: health care worker.

^g^FFP2: filtering face piece 2.

^h^PPM: personal protective measure.

### Distribution and Characteristics of the Studies

**Figure 2:**
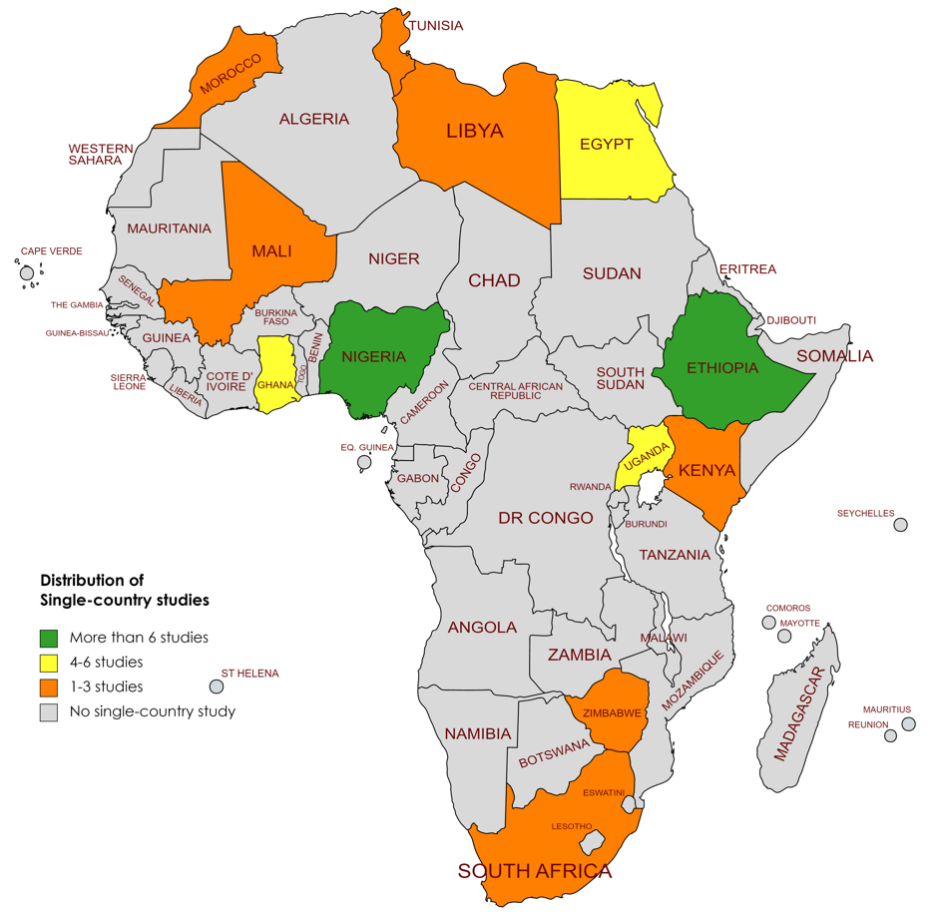
Country distribution of single-country studies. DR: Democratic Republic.

Of the 58 analyzed studies that primarily reported on PPMs against COVID-19 in Africa, 47 (81%) were single-country studies and were conducted in only 12 of the 54 African countries, whereas the remaining 11 (19%) involved multiple countries (Figure 2). Of the single-country studies, 36% (21/58) were from East Africa, 21% (12/58) were from West Africa, 17% (10/58) were from North Africa, 7% (4/58) were from Southern Africa, and none were from Central Africa. Ethiopia (16/58, 28%), Nigeria (7/58, 12%), and Egypt (5/58, 9%) were the top contributors and altogether produced 48% (28/58) of the analyzed studies. Thus, no single-country studies regarding COVID-19 PPMs had been conducted in 42 African countries at the time of our literature search (Figure 2).

The 58 analyzed studies included 51 (88%) quantitative studies, 5 (9%) qualitative studies, and 2 (3%) mixed methods studies, and their sample sizes ranged from 20 to 206,729. The 4 themes were represented as follows: knowledge and perception of PPMs (21/58, 36%), mask use (37/58, 64%), physical and social distancing (17/58, 29%), and handwashing and hand hygiene (19/58, 33%), considering that most studies covered more than one theme. Moreover, 34% (20/58) of the analyzed studies were conducted among health care workers (HCWs), 33% (19/58) were conducted among the general public, 5% (3/58) were conducted among patients with comorbidities, 3% (2/58) were conducted among university students, and 22% (13/58) were conducted among other groups. The studies were published between 2019 and 2022, and their overall quality was generally good, meaning that the included studies satisfied most of the criteria. However, lower scores on item 4 (nonresponse bias) and item 5 (appropriateness of statistical methods used) were noted among several quantitative studies (9/51, 18% and 13/51, 26%, respectively), with a similar trend observed among qualitative studies, as detailed in Multimedia Appendix 2.

### Knowledge and Perception of PPMs and Associated Factors

The results of the knowledge and perception of PPMs and other domains are summarized and presented in Table 3. Among the general public, higher rates of knowledge of COVID-19 preventive measures (>60%) were reported in Western Uganda [18] and Northwest Ethiopia [20]. Higher rates of good attitudes and perceptions were also reported in Western Uganda [18] and Egypt [21], but a lower rate of perceived benefits of preventive measures was reported in Addis Ababa, Ethiopia [22]. Similar findings of good perception were also reported in the Greater Kampala Metropolitan area of Uganda and in other 6 countries (Botswana, Kenya, Malawi, Nigeria, Zambia, and Zimbabwe), where a great majority of the residents believed that face masks were effective against COVID-19 spread and infection [23,24]. However, most individuals in Nigeria believed that COVID-19 was more of a hoax than a reality, and in several countries, preventive measures such as social distancing and face masking were perceived as imported policies that negatively affected their compliance with preventive measures to curb the spread of the disease [25,26].

**Table 3:** Levels and associated factors of personal protective measure (PPM) knowledge and perceptions and the practice of face mask use, social and physical distancing, and hand hygiene.

		Knowledge and perceptions of PPMs	Face mask use	Social and physical distancing	Handwashing and hand hygiene
**General public**					
	Levels	Higher rates (>60%) of knowledge of PPMs [18,20] Higher rates (>60%) of good attitude and perception [18,21,23,24] Lower rates (<60%) of good attitude and perception [22,25,26]	Higher rates (>60%) [24,27,31,32] Lower rate (<60%) [18,20,22,27,29,39]	Higher rate (>60%) information not available Lower rates (<60%) [20-22,28-30,39]	Higher rate (>60%) information not available Lower rates (<60%) [33,37-39]
	Associated factors	Educational level, age, frequency of PPE^a^ practice, and social media exposure [18,23,24]	Gender, age, educational level, marital status, working status, profession, place or community of residence, knowledge and attitude, strictness of containment and health policies, perceived risk and barriers, cues to action, and self-efficacy, among others [22,27,29,32]	Gender, age, educational level, working status, place or community of residence, family size, knowledge and attitude, strictness of containment and health policies, perceived risk and barriers, cues to action, and self-efficacy, among others [20-22,28-30]	Gender, age, educational level, marital status, profession, place or community of residence, knowledge and attitude, exposure to handwashing guidelines, strictness of containment and health policies, type and availability of water sources, and perceived risk and barriers, among others [19,22,29,33]
**Health care workers**					
	Levels	Higher rates (>60%) of knowledge and attitude [40-42] Lower knowledge rate (<50%) [43]	Higher rates (>60%) [49-53] Lower rates (<60%) [41,45-48]	—^b^	Higher rates (>60%) [40,49,52,53] Lower rates (<60%) [45-47]
	Associated factors	Age, practice location, PPE attitude, and perceived threat [42,43]	Gender, educational level, work experience, medical specialty (being a nurse or midwifery professional), hours of work, previous training on COVID-19 prevention and PPE use, perceived risk and attitude toward COVID-19, feedback on safety, having COVID-19 management guidelines, and ease and safety when using standard precautions, among others [44-49,55]	—	Educational level, gender, work experience, medical specialty (being a nurse or midwifery professional), previous training on COVID-19 prevention and PPE use, perceived risk and attitude toward COVID-19, feedback on safety, having COVID-19 management guidelines, and ease and safety when using standard precautions, among others [45-47,49]
**Other groups**					
	Levels	Higher rates of knowledge, good attitude, and perception among university students [64,65], patients with chronic diseases [61], government employees [68], taxi drivers [70,71], and market vendors [72] Poor perception among mothers [66]	Higher rates (>60%) among patients with chronic diseases [61,62], university students [64], government employees [68], patrons of convenience shops [74], and taxi drivers [70] Lower rate (<60%) among medical students [65], children [66], quarantined individuals [67], commuters [69], and shopping centers [73]	Higher rates (>60%) among patients with chronic diseases [61,62], government employees [68], and commuters [69] Lower rate (<60%) information not available	Higher rates (>60%) among patients with chronic diseases [61,62], government employees [68], taxi drivers [71], and bank visitors [75] Lower rate (<60%) among shopping centers [73] and patrons of convenience shops [74]
	Associated factors	Age, educational level, income, and residence (for taxi drivers and government employees) [68,70,71]	Age, gender, knowledge, attitude, field of study (for university students), educational level, employment status, history of having COVID-19, and perceived benefit and susceptibility, among others [62,64-67]	Knowledge and attitude [62]	Age, marital status, knowledge, educational level, attitude, and adherence to infection and control guidelines, among others [62,71,74]

^a^PPE: personal protective equipment.

^b^Not available.

Among African HCWs, higher rates of knowledge and attitude regarding the use of PPE were reported in Ethiopia [40,41] and Egypt [42]. However, a lower knowledge rate of PPE use (<30%) was reported in Nigeria, and in the early stages of the pandemic, a large number of frontline HCWs in Tunisia had not received official training on the correct use of PPE as many believed that they needed additional training [44]. In Egypt, although a substantial proportion (>80%) of house officers (fresh medical graduates doing their 1-year training in different specialties) had good PPE attitudes, <30% had good preparedness and willingness to participate in COVID-19 management and care [42].

Among other groups, only 3% of patients with chronic conditions such as cancer in Egypt, Algeria, and Morocco knew someone who had a COVID-19 infection, but most were worried about contracting the virus [61]. In Ethiopia, although university students had higher rates (>85%) of good attitude toward face mask use, their overall knowledge about mask use was very low (<30%) [64], which contrasts with medical students in Uganda with a better knowledge rate and where close to 70% agreed on the superiority of double masking over single masking for COVID-19 prevention and control [65]. In Nigeria, >50% of mothers perceived masking in children as not an appropriate preventive measure against COVID-19 because of the perceived difficulty in breathing and discomfort among children [66]. Notably, government employees in Ethiopia were reported to have higher rates of good perception of COVID-19 PPMs, whereby approximately 80% perceived consistent mask wearing as highly effective against COVID-19 spread and infection [68]. High COVID-19 vulnerability perception was also reported among commercial drivers in Ghana, mostly among older drivers, who consistently wore face masks and insisted on other persons in their vehicles doing the same [70]. Similar findings were reported among Ethiopian drivers who had good knowledge and positive attitudes [71]. Furthermore, most of the high-risk individuals in Uganda, including market vendors, had received information on face mask use and believed that face masks were protective against COVID-19. Moreover, those who had received information on face mask use were more likely to own face masks and perceive them as protective despite market vendors being more likely to reuse face masks than hospital workers [72].

As possible predictors, educational level, age, income, residence, frequency of PPE practice, PPE attitude, social media exposure, and perceived threat were associated with knowledge and perception of PPMs among the general population, HCWs, and other groups (taxi drivers and government employees) [18,23,24,42,43,68,70,71]. Moreover, practice location was notably significantly associated among HCWs [43] (Table 3).

### Mask Use and Associated Factors

Among the general public, largely lower adherence rates (range 20.3%-59.4%) of face mask use were reported in various African countries, including Uganda, the Democratic Republic of the Congo, Somalia [18,27], and Ethiopia [20,22,29]. Notably, adherence rates to mask use were reported to be higher (>60%) after the lifting of lockdown restrictions and in countries where mask use was mandatory, such as Mozambique [27], Uganda [24], Morocco [31], and South Africa [32]. In addition, reusable cloth masks, which are more cost-beneficial and environmentally friendly, were the most used mask type [27]. Interestingly, in South Africa, the prevalence of close others’ mask wearing was reported to affect mask use, and older adults had poor mask use practices despite having a higher mortality risk [32]. In addition, the prevalence of mask wearing was noted to have increased substantially (50% to 74%) from May 2020 to August 2020 as COVID-19 cases increased and lockdown restrictions were eased, but staying at home, physical distancing, and social distancing decreased [32]. Regarding used mask disposal, poor disposal practices were reported in several African countries, including Morocco, where most threw their used masks and gloves in their house trash or trash bins, posing a transmission risk to sanitary workers or stray animals [31].

Among HCWs, varying rates of PPE use were reported in different regions and countries. Generally, lower rates (<60%) were reported in the Northwest [45], Northeast [46], Eastern [47], and Amhara regions [41] of Ethiopia, as well as in Egypt [48]. Nevertheless, higher rates (>60%) were reported in Nigeria [49], Libya [50], and 3 multicountry surveys [51-53]. The main challenges reported were inadequate PPE and side effects. The lack of PPE and, thus, the reuse of single-use PPE, especially facial protective shields and masks, were reported in several African countries, including Tunisia [44], Ethiopia [40,54], Egypt [48], and Nigeria [55], and in a multicountry survey including 4 North African countries [51].

Several side effects because of the use of PPE were also reported among African HCWs, including skin problems; heat; thirst; pressure areas; headaches; inability to use the bathroom; extreme exhaustion; discomfort; and reduced vision, concentration, and performance during or after wearing PPE [40,44,51,56-60]. Moreover, such side effects were associated with longer shift durations, the frequency of use, and medical specialty [51,58-60], and the most affected body areas from wearing PPE were the hands, the auricular area, the nasal bridge, the cheeks, and the whole face [52]. Notably, the most reported adverse reactions particularly because of using sanitizers were skin dryness, skin irritation, and ocular irritation [40,52]. Moreover, bleach immersion was reported to be highly associated with hand reactions, whereas hand cream use more than twice daily was associated with fewer reactions [60]. In contrast, a recent multicountry survey that included Egypt indicated that >70% of HCWs had all the PPE and protective measures they needed, >60% had been recently educated on COVID-19 infection control, and none of the interviewed HCWs refrained from using face masks [53].

Among other population groups, strict adherence to face mask use in public areas was reported among patients with cancer in a multicountry survey that included Egypt, Algeria, and Morocco [61]. In Ethiopia, >50% of adults with chronic conditions intended to practice and had ever practiced the recommended personal preventive measures against COVID-19 [62]. University students in Ethiopia were reported to have a higher adherence rate (>80%) of mask use [64]. Furthermore, approximately 20% of medical students in Uganda practiced double masking, where the lack of trust in the quality of masks was the most compelling factor for double masking [65]. Nonetheless, excessive sweating, the high cost of face masks, and difficulty in breathing were the major barriers to double masking among these medical students [65]. Moreover, poor adherence to mask use was highlighted among quarantined individuals in Ethiopia, where nearly half of them did not wear a face mask when leaving home [67]. However, high rates (>80%) of mask use were documented among government employees in Ethiopia [68]. Similar findings of high mask use (>70%) were also observed among taxi drivers [70] and patrons of convenience shops in Ghana [74] but with contrasting observations among commuters and in shopping centers, where less compliance with face mask use was reported [69,73].

Adherence to mask use was associated with gender, age, educational level, marital status, working status, profession, place or community of residence, knowledge and attitude, history of having COVID-19, perceived benefit, strictness of containment and health policies, subjective norms, perceived risk, barriers, cues to action, and self-efficacy among the general public, HCWs, and other groups (Table 3) [22,27,29,32,44-49,55,62,64,65,67,70]. Moreover, work experience, medical specialty (being a nurse or midwifery professional), hours of work, previous training on COVID-19 prevention and PPE use, having COVID-19 management guidelines, and ease and safety when using standard precautions were outstanding predictors among HCWs [44-49,55], and the field of study was a strong predictor of mask use among university students [64]. Surprisingly, mask use among children in Nigeria was highly dependent on the mother’s opinions and characteristics, whereby it was associated with the mother’s age, the age of the child, and the parental level of education [66].

### Social and Physical Distancing and Associated Factors

Generally, lower adherence rates (range 18%-59%) of social distancing were reported in various African countries, including Egypt [21], South Africa [28], and Ethiopia [20,22,29,30]. Nonetheless, physical distancing policies disrupted social life and infringed on people’s sociocultural rights, causing adverse socioeconomic and health consequences, especially for low-income urban or suburban slum dwellers [26,34]. Although the imposition of COVID-19 distancing regulations led to a substantial decrease in extrahousehold social contacts (close physical and conversational contacts) in several African countries, including South Africa, there was ongoing contact within intergenerational households, highlighting a potential limitation of social distancing measures in protecting older adults [35]. In contrast, such restrictive policies improved feeding habits through increased meal planning and selection and preparation of healthy foods among residents of various countries [36].

Regarding the implementation and adoption of physical distancing measures, despite the implementation of various mitigation measures, the internally displaced people in Mali still faced several challenges, including the proximity in which internally displaced people live, the lack of toilets and safe water, and the lack of financial resources [38]. Similar findings were reported among prisons in Zimbabwe, where there were several challenges in the adoption of COVID-19 PPMs, such as severe congestion, interrupted water supply, outdated infrastructure, and inadequate hygiene and sanitation [39]. Moreover, although prisoners had adequate COVID-19 awareness and prison health professionals received training on COVID-19 control measures, PPE supply was inadequate, with no routine COVID-19 testing in place beyond thermal scanning; isolation measures were compromised by accommodation capacity issues; and social distancing was impossible during meals and at night [39].

Among other population groups, strict adherence to social and physical distancing was documented among patients with cancer, and most preferred web-based medical appointments over regular visits. In addition, some adopted healthier diets, used dietary supplements, and recited the Quran or supplications [61]. Similar findings of good practice of social distancing were also reported among patients with chronic conditions in Ethiopia [62]. Moreover, in Egypt, patients preferred teledermatology services to the usual physical clinic visits as they perceived them as reliable and safe during the pandemic [63]. Government employees in Ethiopia also had higher rates (>80%) of good practice of physical distancing [68], and the same applied to commuters in Ghana, who had high compliance rates with social distancing guidelines [69].

Adherence to social and physical distancing was associated with gender, age, educational level, working status, place or community of residence, family size, knowledge and attitude, strictness of containment and health policies, perceived risk and barriers, cues to action, and self-efficacy among the general public and other groups (patients with chronic diseases; Table 3) [20-22,28-30,62].

### Handwashing and Hand Hygiene and Associated Factors

Regarding community adherence to hand hygiene, lower rates (<60%) were reported in 12 sub-Saharan countries, where the likelihood of handwashing mainly varied with the level of concern about COVID-19 [33]. In resource-restricted settings, a recent study indicated that >60% of the slum dwellers in Nairobi, Kenya, had limited water, sanitation, and hygiene facility accessibility and opportunity, making adherence to COVID-19 PPMs impossible [37].

Concerning hand hygiene adherence among African HCWs, varying rates were reported in different regions and countries. Generally, lower rates (<60%) were reported in the Northwest [45], Northeast [46], and Eastern [47] regions of Ethiopia. Nevertheless, higher rates (>60%) were reported in Nigeria [49], Southwest Ethiopia [40], and 2 multicountry surveys [52,53].

Regarding other groups, strict adherence to proper hand hygiene was reported among patients with chronic conditions in various African countries, including Egypt, Algeria, Morocco [61], and Ethiopia [62]. A similar observation was made among government employees [68] and taxi drivers [71] in Ethiopia, both of whom had higher rates (>60%) of frequent handwashing and hand hygiene as a means of protection against COVID-19. However, poor adherence to COVID-19 safety protocols at shopping centers in Ghana was reported, whereby, although shops complied with providing handwashing facilities, most of the customers did not practice handwashing before entering the shops and did not wear face masks during shopping, and neither did the shop attendants [73]. Similarly, a very low rate (10%) of appropriate handwashing was reported among patrons of convenience shops in Accra, Ghana [74]. In contrast, an increase in proper handwashing performance was reported among bank visitors in Ethiopia after watching a handwashing demonstration [75].

Handwashing and hand hygiene during the COVID-19 pandemic was associated with gender, age, educational level, marital status, profession, place or community of residence, knowledge and attitude, exposure and adherence to handwashing guidelines, strictness of containment and health policies, type and availability of water sources, and perceived risk and barriers among the general public, HCWs, and other groups (Table 3) [19,22,29,33,45-47,49,62,71,74,75]. Moreover, work experience, medical specialty (being a nurse or midwifery professional), previous training on COVID-19 prevention and PPE use, feedback on safety, having COVID-19 management guidelines, and ease and safety when using standard precautions were notable predictors among HCWs [45-47,49].

## Discussion

### Principal Findings

To our knowledge, this is the first systematic review to evaluate PPMs against COVID-19 among various population groups in Africa. This systematic review has important implications as it reflects cognitive behavioral issues (knowledge and practice) regarding PPMs in some African countries during an infectious disease outbreak. Future outbreaks or waves of COVID-19 may force people to use PPMs again. The review used a multidimensional approach involving the systematic evaluation of evidence based on region, country, and population group. Moreover, comprehensive coverage of the literature was attained, and a reproducible search methodology was applied using a predefined framework, all of which are strengths of this review.

Among the general community, the review showed varying levels of knowledge, attitudes, and perceptions, which in turn influenced the practice levels of and compliance with COVID-19 PPMs, especially face mask use, hand hygiene, and physical and social distancing. This finding is in agreement with a previous study from sub-Saharan Africa [15], and similar findings have been reported in other regions where communities’ cognition directly affected the practice and uptake of COVID-19 PPMs [76]. Nonetheless, the observed difference in the practice and adherence to PPMs across African countries may be due to the differences in COVID-19 control policies, income (gross domestic product), and the situation of the pandemic among the countries. Notably, the compliance rates of face mask use reported in most African communities were generally lower compared with those reported in studies from high-income countries [77,78]. This may partly be explained by the inability to afford to buy face masks and the differences in the strictness of such preventive measures [22,29,79]. Nevertheless, poor adherence to face mask use was also reported in some high-income countries such as Australia, Norway, and Sweden [80], the reasons for which may be other than just the inability to afford face masks. Moreover, lower rates of handwashing and hand hygiene were also reported in several African communities, especially among low-income urban and slum dwellers. This was partly due to a lack of safe and clean water in slum communities [37]. Moreover, buying soap or hand sanitizers was an additional financial constraint for low-income urban dwellers and, thus, may be seen as a luxury.

The results indicate a reduction in the rates of PPM practice (mainly mask use and social and physical distancing) noted in several African countries following the lifting of restrictive lockdown measures and the rollout of COVID-19 vaccination programs. This can be partly explained by pandemic fatigue as more people become demotivated and exhausted to follow the recommended infection prevention and control measures owing to the prolonged impact and existence of COVID-19 [81]. As COVID-19 PPMs complement the vaccination protective advantage, this implies a need for continued community sensitization and education programs to rectify the reluctance to practice PPMs amid the relaxation of preventive restrictions. Moreover, prompt management of infodemics in the current and future infectious outbreaks is needed to address the misinformation about PPMs [82].

Among African HCWs, generally good knowledge of PPE use was reported but with varying levels of practicing PPMs, and the low practice rates were attributed mainly to the lack of PPE and the side effects of prolonged PPE use. With HCWs being at the frontline of screening and managing suspects and patients with COVID-19, the lack of PPE increases the risk of infection when doing their work. Nonetheless, the lack of PPE has also been documented in other countries and regions outside Africa [83]. Furthermore, this review showed that most patients with comorbidities in Africa reported strict adherence to COVID-19 PPMs, which may be due to their perceived high vulnerability to COVID-19 infection and complications. Other studies outside Africa have reported similar findings among patients with comorbidities [84,85].

The study findings show that several cognitive (including knowledge, attitude, and perception), demographic, and socioeconomic factors were associated with the practice of and compliance with COVID-19 PPMs among African communities. COVID-19 being a newly evolving disease with varying cross-cutting impacts implies a need for consideration of such cognitive, demographic, and socioeconomic differences in the design of targeted response measures against the pandemic. Nonetheless, similar findings on the association of sociodemographics with the practice of COVID-19 PPMs have been reported in other regions outside Africa [80].

This review has some practical recommendations for improving COVID-19 control programs in Africa. Efforts are needed to improve the local capacity to produce and supply PPE, especially to HCWs, as the lack of PPE was the main barrier to PPE use. In the early phase of the pandemic, most countries were overwhelmed by the increased demand for PPE, which disrupted the global supply chain, and this had dire consequences for countries with inadequate local manufacturing and supply capacity [83]. In addition, providing free or subsidized face masks and soap, especially to low-income earners, would be a helpful strategy for improving PPM practice and adherence. Moreover, the consideration of vulnerable groups such as low-income urban dwellers and internally displaced people and targeted responses tailored to their socioeconomic dynamics are paramount for effective pandemic control programs. Knowledge and perception influenced the practice of PPMs, implying a need for continuous infodemic management, community education, and sensitization, and this should be tailored to address the existing misconceptions and barriers to PPM adherence. Notably, although several of the analyzed studies (11/58, 19%) evaluated the association between age and the practice of COVID-19 PPMs and showed varying rates and results, no single study focused on exploring COVID-19 PPMs among the older adult population of Africa. Given the known vulnerability of older people to severe COVID-19, efforts are needed to explore this special group to help fully understand their behavioral response to the pandemic, which is vital for guiding targeted responses.

The review also reveals substantial inequalities in terms of research output from different regions of Africa, with PPM studies mostly coming from East, West, and North Africa and only 3 countries (Ethiopia, Nigeria, and Egypt) producing >40% (28/58, 48%) of all the studies. This finding coincides with the study by Nwagbara et al [15], which showed the dominance of East and West Africa in COVID-19 research. The high PPM research output from North and West Africa could be because they were the first regions to record COVID-19 cases in the continent [86]. Although South Africa is known to lead African research with sound and more vibrant research institutions in the continent [14], its contribution to COVID-19 PPM research is far lower, as indicated by the study results. Regarding other African countries, the observed pattern may be explained by the differences in research capabilities and resources. Nonetheless, such research inequalities pose gaps in understanding how such countries and regions respond to the COVID-19 pandemic. This implies a need for more focus, funding, and involvement in behavioral health research, which is as important as clinical research and vital in guiding evidence-based and country-specific or tailored policies and responses in addressing the dynamics of the current COVID-19 pandemic.

### Limitations

This systematic review has some limitations. Although we used a comprehensive keyword search strategy, some relevant studies might have been missed as only 3 databases and only English-language articles were considered. In addition, we did not consider gray literature and preprints in this review; thus, they should be considered in future or updated reviews on PPM practice for a more comprehensive search. Although a comprehensive search was performed, no relevant studies were found from 42 of the 54 African countries; thus, the findings might not provide a comprehensive picture of the knowledge and practice of PPMs across the entire continent. Moreover, the findings and conclusions of this review are based on studies that were mostly web-based surveys, which, although this was inevitable because of the restrictive preventive measures and lockdowns, are prone to selection bias based on internet accessibility. Owing to the self-report nature of these surveys, recall and social desirability bias cannot be overlooked. Moreover, assessments of statistical analyses of associations with the practice of COVID-19 preventive measures, as well as meta-analyses, were not performed as these were not the main focus of this review. Despite these limitations, this study provides valuable insights into the facilitators of and barriers to the practice of PPMs in Africa.

### Conclusions

This review evaluated the knowledge and practice of COVID-19 PPMs in African countries. The findings, conclusions, and recommendations of this review specifically apply to the included countries and, thus, should be interpreted with caution. The results indicate that African communities, including various population groups, have varying levels of practice and compliance with COVID-19 PPMs, with the lack of PPE (mainly face masks) and side effects of PPE use being the major reasons for poor compliance, especially among HCWs. In addition, various cognitive, sociodemographic, and economic factors were associated with the practice of COVID-19 PPMs. Therefore, this review highlights a need for enhancing the local capacity to produce and supply PPE. The consideration of various cognitive, demographic, and socioeconomic differences, with extra focus on low-income urban dwellers and those who are less advantaged, is vital for inclusive and more effective strategies against the pandemic. Moreover, more focus, involvement, and funding of community behavioral (including protective measures) research is needed to fully understand and address the dynamics of the current pandemic in Africa.

## Appendix

Multimedia Appendix 1 Filled-in PRISMA (Preferred Reporting Items for Systematic Reviews and Meta-Analyses) checklist.

Multimedia Appendix 2 Quality assessment of the included studies using the Mixed Methods Appraisal Tool—version 2018.

## Abbreviations

HCW: health care worker
PPE: personal protective equipment
PPM: personal protective measure
PRISMA: Preferred Reporting Items for Systematic Reviews and Meta-Analyses

## Data Availability

All data generated or analyzed during this study are included in this published paper (and its supplementary information files).
