# Supplementary material for: Knowledge and Practice of Personal Protective Measures Against COVID-19 in Africa: Systematic Review
Source: JMIR Public Health Surveill. 2023 May 16;9:e44051. doi: 10.2196/44051 (PMC10198719; doi:10.2196/44051)
Supplement: Multimedia Appendix 2 [file publichealth_v9i1e44051_app2.docx]

**Multimedia Appendix 2: Quality assessment of included studies by Mixed Methods Appraisal Tool (MMAT) – Version 2018**

| **Criteria** |  | QND  n=51  (%) | QL  n=5  (%) | MM  n=2  (%) |
| --- | --- | --- | --- | --- |
| QND1. Is the sampling strategy relevant to address the research question? | Yes | 94 |  |  |
|  | No | 2 |  |  |
|  | Can’t tell | 4 |  |  |
| QND2. Is the sample representative of the target population? | Yes | 90 |  |  |
|  | No | 2 |  |  |
|  | Can’t tell | 8 |  |  |
| QND3. Are measurements appropriate (clear origin, or validity known, or standard instrument)? | Yes | 82 |  |  |
|  | No | 12 |  |  |
|  | Can’t tell | 6 |  |  |
| QND4. Is the risk of non-response bias low? | Yes | 50 |  |  |
|  | No | 18 |  |  |
|  | Can’t tell | 32 |  |  |
| QNR5. Is the statistical analysis appropriate to answer the research question? | Yes | 64 |  |  |
|  | No | 26 |  |  |
|  | Can’t tell | 10 |  |  |
| QL1. Is the qualitative approach appropriate to answer the research question? | Yes |  | 100 |  |
|  | No |  | 0 |  |
|  | Can’t tell |  | 0 |  |
| QL2. Are the qualitative data collection methods adequate to address the research question? | Yes |  | 100 |  |
|  | No |  | 0 |  |
|  | Can’t tell |  | 0 |  |
| QL3. Are the findings adequately derived from the data? | Yes |  | 100 |  |
|  | No |  | 0 |  |
|  | Can’t tell |  | 0 |  |
| QL4. Is the interpretation of results sufficiently substantiated by data? | Yes |  | 60 |  |
|  | No |  | 0 |  |
|  | Can’t tell |  | 40 |  |
| QL5. Is there coherence between qualitative data sources, collection, analysis and interpretation? | Yes |  | 40 |  |
|  | No |  | 0 |  |
|  | Can’t tell |  | 60 |  |
| MM1. Is there an adequate rationale for using a mixed methods design to address the research question? | Yes |  |  | 100 |
|  | No |  |  | 0 |
|  | Can’t tell |  |  | 0 |
| MM2. Are the different components of the study effectively integrated to answer the research question? | Yes |  |  | 100 |
|  | No |  |  | 0 |
|  | Can’t tell |  |  | 0 |
| MM3. Are the outputs of the integration of qualitative and quantitative components adequately interpreted? | Yes |  |  | 100 |
|  | No |  |  | 0 |
|  | Can’t tell |  |  | 0 |
| MM4. Are divergences and inconsistencies between quantitative and qualitative results adequately addressed? | Yes |  |  | 100 |
|  | No |  |  | 0 |
|  | Can’t tell |  |  | 0 |
| MM5. Do the different components of the study adhere to the quality criteria of each tradition of the methods involved? | Yes |  |  | 100 |
|  | No |  |  | 0 |
|  | Can’t tell |  |  | 0 |

QND: quantitative descriptive studies; QL: qualitative studies; MM: mixed-method studies

The MMAT assesses the quality of qualitative, quantitative, and mixed methods studies. It focuses on methodological criteria and includes five core quality criteria for each of the following five categories of study designs: (a) qualitative, (b) randomized controlled, (c) nonrandomized, (d) quantitative descriptive, and (e) mixed methods. The tool uses three response options to rate each article: 'Yes' meaning the criterion is met, 'No' meaning the criterion is not met, and 'Can't tell' when there is not enough information in the paper to judge if the criterion is met or not. After rating all the studies in each study design, a summary in terms of perentage is given for each of the three respnse options.
